# Femoral head mosaicplasty by direct anterior approach for an osteochondral impaction without performing surgical hip dislocation

**DOI:** 10.1051/sicotj/2021014

**Published:** 2021-03-26

**Authors:** Remy Coulomb, Abdullah Alrubaie, Vincent Haignière, Pascal Kouyoumdjian

**Affiliations:** 1 Department of Orthopedic and Traumatology Surgery, CHU Nîmes, University Montpellier 1 34000 Nîmes France; 2 Université Montpellier 1 2 Rue de l’École de Médecine 34090 Montpellier France

**Keywords:** Hip arthroplasty, Mosaicplasty, Femoral head fracture, direct anterior approach, OATS

## Abstract

Several surgical methods exist for the treatment of osteochondral lesions of the femoral head. They include osteochondral allograft transfer, femoral osteotomy, microfracture, autologous chondrocyte implantation, and hip arthroplasty. Mosaicplasty is a surgical method in which cylindrical plugs of bone and cartilage are transferred from a donor site to tunnels drilled into the bone and cartilage defects. This paper discusses the use of mosaicplasty by a direct anterior approach without dislocation in the treatment of an acute femoral head osteochondral impaction in a young patient.

## Background

Traumatic hip dislocations often lead to damage to the acetabular rim or femoral head [1]. Cases of isolated traumatic femoral head osteochondral lesions are rare, especially for young patients. Conservative treatments for such cases often yield unsatisfactory results and may lead to post-traumatic osteoarthritis [2]. Alternative methods such as total hip arthroplasty yield acceptable functional results but are not suitable for young patients [3, 4]. On the other hand, micro fracturing gives good short-term results due to delayed osteoarthritis. It does not, however, result in hyaline cartilage formation, but instead, forms low-quality fibrocartilage that later contributes to osteoarthritis [5, 6]. Chondrocyte transplantation is a promising surgical technique but is only limited to the thickness of the articular cartilage [7]. Mosaicplasty offers an option that traverses the osteochondral thickness and allows for hyaline formation [8]. This helps in maintaining the integrity of the articular surface that is an important outcome for young active patients. However, femoral head mosaicplasty involves surgical dislocation with a risk of avascular necrosis [9–11]. The purpose of this article is to present the surgical technique and early outcomes for a case in a 16-year-old female with osteochondral femoral head impaction who underwent mosaicplasty by direct anterior approach without performing surgical hip dislocation.

## Case report

### Case and primary care

**Figure 1:**
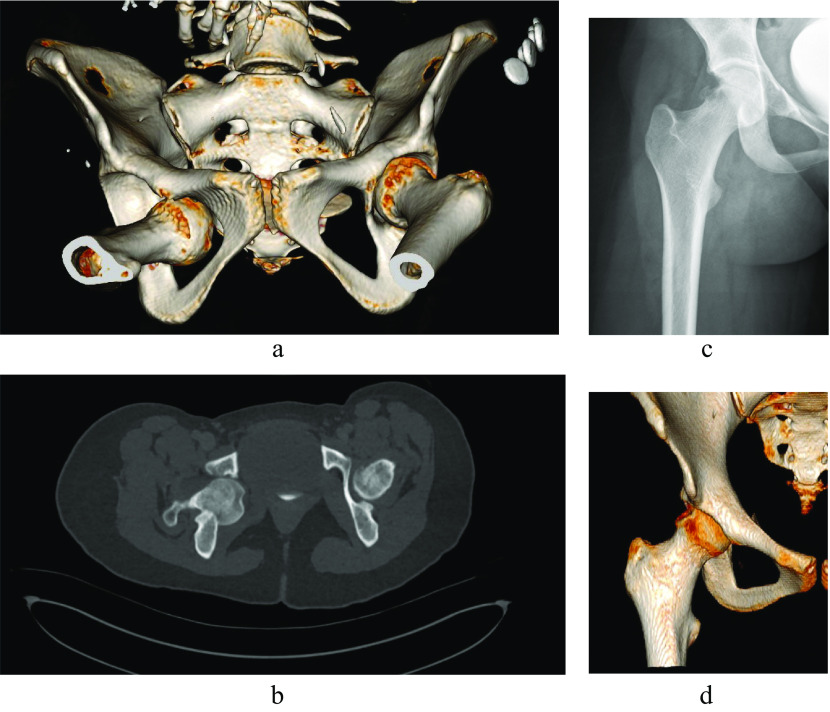
Right hip obturator dislocation before (a, b) and after closed reduction (c, d).

A 16-year-old girl was transferred to the emergency department with a history of a motor vehicle accident. A full-body CT showed right hip obturator dislocation with femoral head impaction and acetabular teardrop fracture; no other associated traumatic injuries were detected. The patient had closed reduction performed under general anesthesia. An X-ray showed a good reduction with an important osteochondral impaction and a wide lateral femoroacetabular joint space. However, a CT scan, showed an osteochondral defect on the femoral head measuring approximately 3 × 2 cm (Figure 1).

### Surgical management

**Figure 2:**
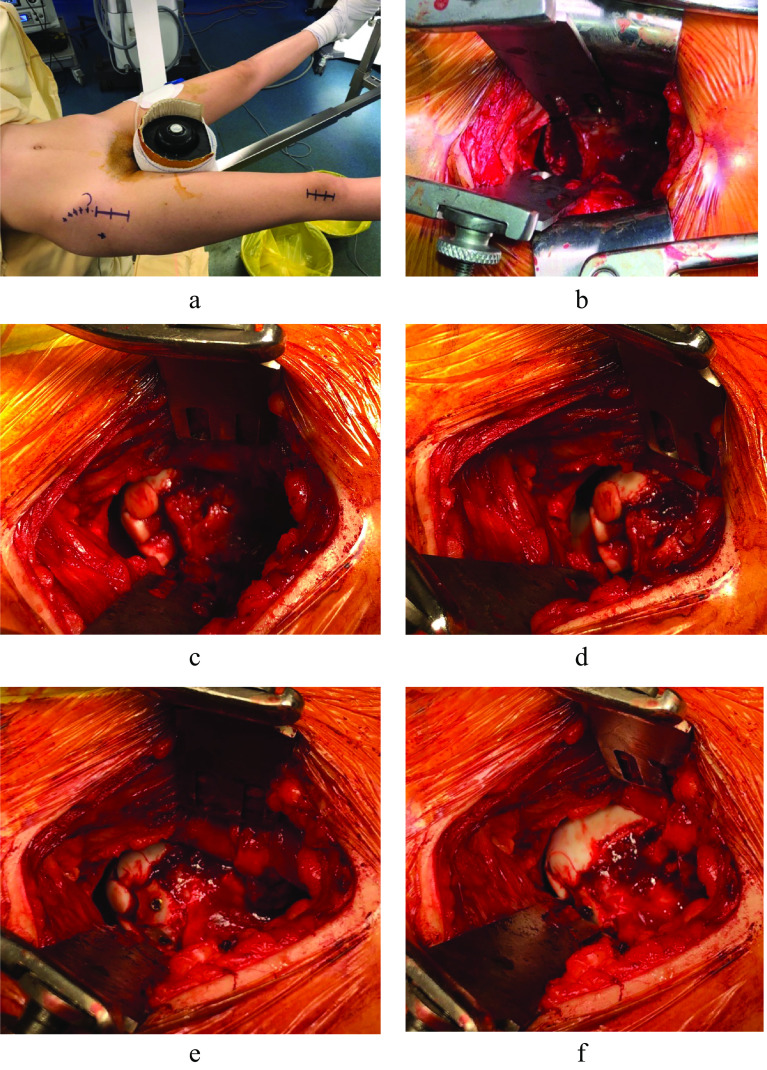
Intra-operative images of the right hip, demonstrating patient positioning (a), osteochondral lesion of the femoral head (b), femoral head after insertion of three osteochondral plugs (c, d), and filling of the non-weight-bearing surface with a screwed iliac crest autograft (e, f).

The entire surgical operation was under general anesthesia with the patient lying supine on a traction orthopedic table (Figure 2a). The hip was approached anteriorly using a modified Hueter technique with a standard transverse incision of 8 cm. A T-shaped capsulotomy was done taking care to maintain the integrity of the labrum and avoid damage to the anterior circumflex femoral artery. Traction was applied to obtain a joint space 1 cm wide. With this approach, we can identify the anatomical structures visually and use retractors to expose the anterolateral zone to facilitate our entry to the femoral osteochondral defect (Figure 2b). Debridement of the site was performed by a short anterolateral approach in traction until the subchondral bone was visible and the edges of the cartilage were stable. The anterolateral approach was required to prepare perpendicularly the receiving area of the femoral head. Cylindrical osteochondral plugs were harvested from three 10 mm non-weight bearing areas of the femoral trochlear surfaces, two lateral aspects, and one medial aspect. The traction was released during osteochondral plugs extraction. The transfer was done using the Osteochondral Autograft Transfer System technique (OATS) (Arthrex, Naples, Florida). The donor plugs were transplanted by the anterolateral approach to the donor site, starting posteriorly toward the anterior area of the defect, while rotating the hip and ensuring orthogonal position both visually under traction and by using C-arm fluoroscopy. The whole procedure was carried out with caution to recreate the curvature of the femoral head and maintain the cartilage surface level (Figures 2c and 2d). An additional space resulting in the non-weight-bearing areas was filled using a cancellous bone autograft from the iliac crest; it was fixed using the AutoFIX Cannulated Screw System (AutoFIX System, Stryker) of a 4 mm diameter and 26 mm length (Figures 2e and 2f). The total traction time was less than 2 h.

### Post-operative care and follow-up

The patient was put on epidural analgesia for 3 days post-operation to manage the surgical pain. Toe-touch weight bearing on crutches was then started on the operated limb and continued for 6 weeks. Throughout the period, deep vein thrombosis prophylaxis was done using low molecular weight heparin (Clexane). Progress was then made for a total weight bearing on the surgical limb, as tolerated by the patient, and under the supervision of the orthopedic surgeon.

The same primary team followed up the patient a year later. The Harris, Oxford, and Non-Arthritric hip scores were 93, 14, and 90/100 respectively. The patient’s satisfaction was good with only slight knee residual pain and stiffness. On clinical examination, she was standing and walking normally with an extension/flexion, abduction/adduction, and abduction/ adduction of 10°/120°, 40°/30°, and 60°/30° respectively.

**Figure 3:**
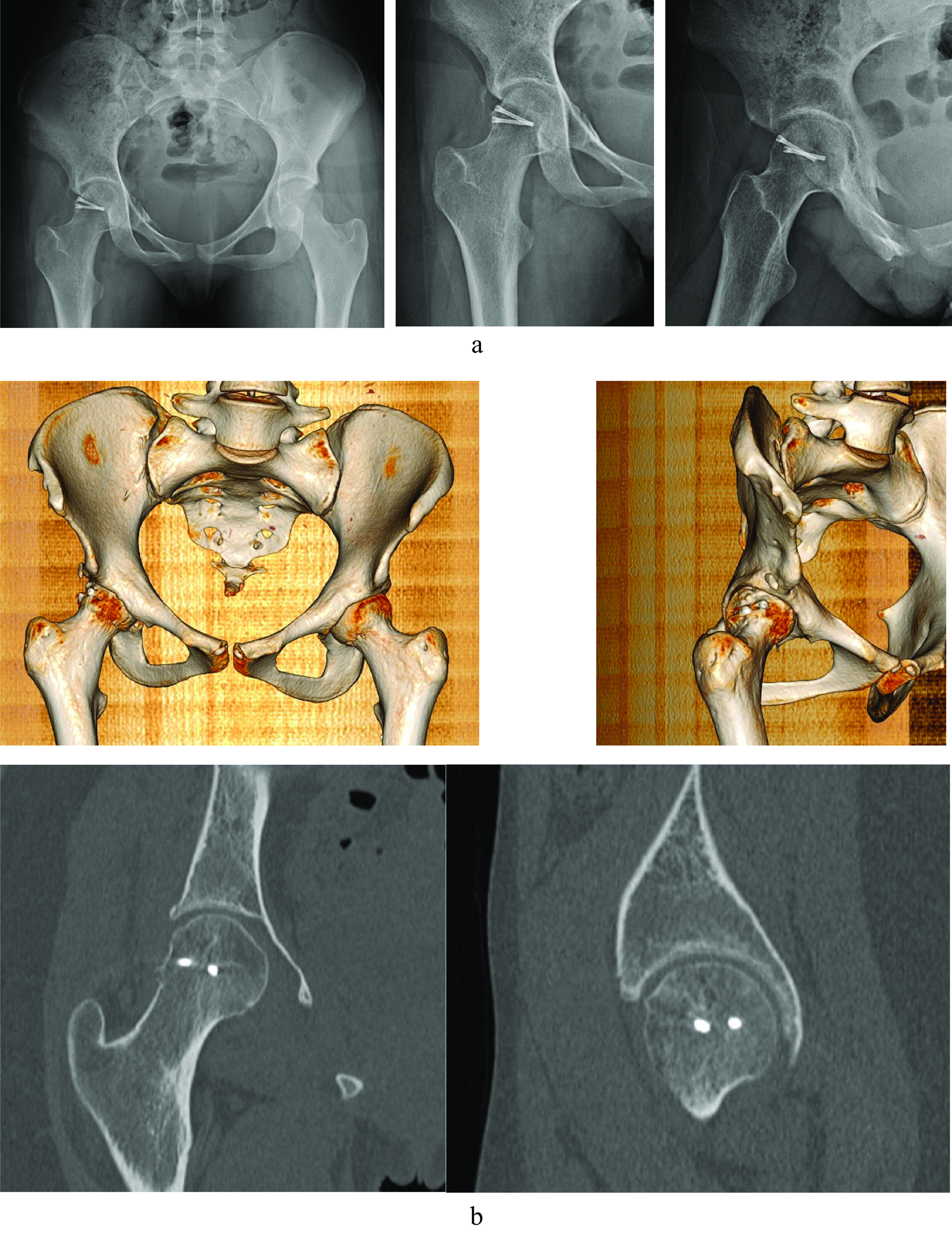
X-ray image of the right hip 3 months after surgery (a) *left*: pelvic AP, *middle*: right AP axial, *right*: external and internal oblique view (b) CT scan 1 year after surgery demonstrating both femoral head screws and a slightly irregular surface with good joint space at the upper part of the femoral head.

X-rays showed that the hip joint was in place and the femoral head was slightly curved (Figure 3a). A CT-scan after 1-year follow-up demonstrated a good integration of the osteochondral plugs with a minimal chondral step-off, and no aseptic osteonecrosis of the femoral head or hip osteoarthritis (Figure 3b).

## Discussion

In this case, we treated a large traumatic superolateral osteochondral lesion of the femoral head using OATS by direct anterior approach without dislocation.

The common approach to access the femoral head for hip preservation surgery is the Gibson approach [12]. Nevertheless, the Gibson surgical approach needs piriformis muscle release and a digastric trochanteric osteotomy to exposed the femoral head. Complications reported were sciatic nerve injury, superior gluteal artery injury, delayed trochanteric union, and heterotopic bone formation [13]. This suggests a slower recovery, with a risk of delayed normal walking and possible temporary limping. The direct anterior approach was developed to take advantage of muscular neutral planes that could enhance patient satisfaction and functional outcome after hip arthroplasty [14]. Therefore, with excellent knowledge of this approach to the hip joint, it seemed possible to achieve an osteochondral transfer to the lateral part of the femoral head without dislocation. The good clinical results can probably be due to non-dislocation and muscle-sparing of the direct anterior approach.

Although the risks of complication following surgical dislocation of the native hip have the reputation of being common, a recent prospective study shows a low complication rate [15]. The overall complication rate was 9% in this retrospective study of 334 hips; this includes those that were asymptomatic that required no treatment. In a recent study, authors proposed femoral head mosaicplasty by minimally invasive anterior approach with hip dislocation [11]. They found no specific complications to the direct anterior approach or to the dislocation. It is obvious that in our case, the location and type of the lesion allowed us to avoid dislocation and to position OATS under traction only. However, it had the drawbacks of prolonged traction times, no access to the lateral and posterior parts of the hip joint, labrum hindrance to access of the medial part of the lesion, possible injury to the lateral femoral cutaneous nerve, and an uncomfortable and possibly injurious surgical position [16].

The morbidity of the donor site is common, particularly if it is not the same pathology localization. At the last follow-up, the patient experienced pain at the donor site, which is reminiscent of the morbidity of the donor site. Several authors have described the OATS harvest on the ipsilateral hip, which reduces the morbidity of donor site [11, 17]. In our case, we decided to use the ipsilateral knee for the harvesting of the osteochondral plots because of the post-traumatic situation of the hip.

Mosaicplasty leads to chondrogenesis to give hyaline cartilage that links the plugs and ensures smooth convexity of the head of the femur. Very few studies involving the use of mosaicplasty in the large femoral head with defects greater than 4 cm^2^ have been done [1]. In our case, the defect was greater than 4 cm^2^ and we used three 10 mm OATS disposed of in one lateral raw. The use of large mosaicplasty plugs yielded good results because they provided an excellent fit into the large femoral defect [18]. Kordas et al. [19] suggests that grafts of greater diameter are more stable and row or circular implantation do not affect primary stability. However, multiple grafts may not be as stable as single grafts in the initial period after transplantation. In our case, considering the spherical shape of the femoral head and the uncovered lateral part of the plugs, a complementary screwed autologous graft was performed to gain stability.

## Conclusion

Femoral head mosaicplasty by direct anterior approach without dislocation is a rational and appropriate solution in patients with femoral head lateral impaction. With this report, we hope to add another technique to the already existing yet little experience of femoral head mosaicplasty.

## Conflicts of interest

The author(s) declared the following potential conflicts of interest with respect to the research, authorship, and/or publication of this article: PK: is a paid consultant for Stryker and Lepine. No funding was received for the present study. All other authors declare that there is no conflict of interest.
